# Lead-free hybrid perovskite N(CH_3_)_4_SnI_3_ with robust ferroelectricity induced by large and non-polar N(CH_3_)_4_^+^ molecular cation

**DOI:** 10.1038/s41467-021-20889-y

**Published:** 2021-01-27

**Authors:** Hai Wei, Yali Yang, Shiyou Chen, H. J. Xiang

**Affiliations:** 1 Key Laboratory of Computational Physical Sciences (Ministry of Education), State Key Laboratory of Surface Physics, and Department of Physics, Fudan University, Shanghai, 200433 China; 2 Collaborative Innovation Center of Advanced Microstructures, Nanjing, 210093 China; 3 Key Laboratory of Polar Materials and Devices (Ministry of Education), East China Normal University, Shanghai, 200241 China

**Keywords:** Solar cells, Ferroelectrics and multiferroics

## Abstract

The ferroelectricity in the hybrid perovskite CH_3_NH_3_PbI_3_ is under debate because it results from the polar molecular cation CH_3_NH_3_^+^ while the molecular orientation was reported to be random. Here we predict that a Pb-free hybrid perovskite N(CH_3_)_4_SnI_3_ with non-polar molecular cation N(CH_3_)_4_^+^ has strong ferroelectricity with a spontaneous polarization of 16.13 μC cm^−2^. The large polarization results from the distortion of SnI_6_ octahedron induced by the large N(CH_3_)_4_^+^ and is independent of the molecular orientation, so the ferroelectricity is robust. The ferroelectric *R3m* perovskite structure of N(CH_3_)_4_SnI_3_ can be synthesized as the ground state under a hydrostatic pressure over 3 GPa and remains stable under ambient pressure. Given the strong ferroelectricity, good stability and high visible-light absorption, N(CH_3_)_4_SnI_3_ may be an ideal light-absorber semiconductor for high-efficiency solar cells because its ferroelectric polarization can facilitate electron-hole separation and produce large bulk photovoltaic effect, making the design of homogeneous bulk photovoltaic devices possible.

## Abstract (web summary)

The ferroelectricity in hybrid perovskite CH3NH3PbI3 is under debate. Here, the authors predict a Pb-free hybrid perovskite N(CH3)4SnI3 with non-polar molecular cation N(CH3)4^+^ showing strong ferroelectricity, good stability, and high visible-light absorption, ideal for high-efficiency solar cells.

## Introduction

The organic–inorganic hybrid perovskites (OIHPs) drew intensive attention in the past decade as the light-absorber material in the all-solid-state solar cells and the power conversion efficiency (PCE) of the solar cells based on the hybrid perovskites such as CH_3_NH_3_PbI_3_ or NH_2_CHNH_2_PbI_3_ is now as high as 25.2%^1–5^, which is getting closer to that of the monocrystalline silicon (band gap *E*_g_ = 1.2 eV, PCE = 26%)^6^, and higher than that of the commercialized thin-film solar cells based on CdTe (*E*_g_ = 1.5 eV) and CuIn_*x*_Ga_1−*x*_Se_2_ (*E*_g_ = 1.0–1.7 eV, PCE = 20%)^7,8^. The OIHPs have a chemical formula ABX_3_, where *A* = organic molecule cation, *B* = inorganic cation (Pb_2_^+^ or Sn_2_^+^), and *X* = halide anion (Cl^−^, I^−^, Br^−^, or their mixture). One unexpected advantage of the hybrid perovskites as the light-absorber material in solar cells is that it may have ferroelectricity, and the ferroelectric polarization can enhance the electron–hole separation and therefore prevent their recombination^9^. Furthermore, the bulk Rashba effect in the ferroelectric material may also slow down the electron–hole recombination^10^.

Although the ferroelectricity of CH_3_NH_3_PbI_3_ was studied both experimentally and theoretically, the evidence of its ferroelectricity is still under debate and not robust^11,12^. For hybrid perovskites such as CH_3_NH_3_PbI_3_ and [(CH_3_)_3_NCH_2_I]PbI_3_, the A site is occupied by polar organic molecular cations, which can produce spontaneous polarization if the orientation of the polar molecular cations is ordered along a certain direction. However, recent molecular dynamics simulations showed that the polar A-site molecular cations are rotating and their orientation is random at room temperature, so the ferroelectricity should be very weak, or appear in a certain area but disappear quickly. Strong ferroelectricity can exist only after a disorder–order transition occurs for the polar A-site molecule cation in the whole thin film, but it is very difficult to control the molecular orientation. Besides the polarization induced by A-site molecular cations, there are some debates on whether the ferroelectricity of OIHPs can result from the conventional structural distortion induced by B-site ion (displacive mechanism)^13^. This mechanism is independent of the polarity and orientation of A-site molecular cations, so the ferroelectricity can be robust and can be induced in OIHPs with non-polar molecular cation on A-site. Unfortunately, first-principles calculations showed that the polarization induced by the B-site ion displacement is very weak in CH_3_NH_3_PbI_3_, and OIHPs with non-polar molecular cation on A site have seldom been reported to have strong ferroelectricity so far^14^. Since there is no OIHPs with both robust ferroelectricity and suitable band gaps as the light-absorber materials in solar cells, it has not yet been achieved to enhance the electron–hole separation and increase PCE by introducing robust ferroelectric polarization in the hybrid perovskite solar cells.

In this work, we find unexpectedly that a Pb-free hybrid perovskite N(CH_3_)_4_SnI_3_ with the non-polar molecular cation N(CH_3_)_4_^+^ at A site and *R3m* structure can have robust and strong ferroelectricity with an intrinsic spontaneous polarization as large as 16.13 μC cm^−2^. The large polarization results from the displacement of Sn^2+^ cation from the center of SnI_6_ octahedron, rather than the polar molecular cation, so it is robust and unrelated to the orientation of the non-polar molecular cation N(CH_3_)_4_^+^. The band gap of N(CH_3_)_4_SnI_3_ is 2.12 eV and can be tuned effectively from 2.12 to 1.0 eV by applying a 3 GPa hydrostatic pressure, and its optical absorption coefficients for visible light are high. So it can be a potential high-efficiency light-absorber semiconductor in solar cells. The stability calculation showed that the *R3m* structure is the ground state of N(CH_3_)_4_SnI_3_ under a 3 GPa hydrostatic pressure, and the phonon dispersion calculations showed that the *R3m* structure is dynamically stable and can remain stable even after the pressure is released. Therefore, the *R3m* N(CH_3_)_4_SnI_3_ can be synthesized under high pressure and then used as the stable light-absorber material in solar cells under the ambient conditions. As far as we know, N(CH_3_)_4_SnI_3_ is a hybrid perovskite with both strong ferroelectricity and suitable band gap for the visible light absorption, so it offers an ideal material candidate for fabricating stable and Pb-free hybrid perovskite solar cells with a high efficiency enhanced by ferroelectricity.

## Results

Since the ferroelectricity is sensitive to the orientation of molecular cation in hybrid perovskites such as CH_3_NH_3_PbI_3_ with a polar molecular cation at A site, its ferroelectricity may be weak and not stable. In this study, we intend to search for hybrid perovskites with stable and strong ferroelectric polarization, so we focus on the hybrid perovskites with non-polar molecular cations. In the well-studied ferroelectric ABO_3_ perovskites, it had been demonstrated that a large A-site cation can induce large structural distortion and thus strong polarization. Therefore, our search is further focused on the large and non-polar A-site molecular cations, among which the N(CH_3_)_4_^+^ is obviously an ideal candidate, so we expect that N(CH_3_)_4_SnI_3_ may be potential hybrid perovskite with strong ferroelectricity. In the following sections we will discuss the stability and ferroelectricity of N(CH_3_)_4_SnI_3_.

### Structural stability under zero and finite pressure

**Fig. 1:**
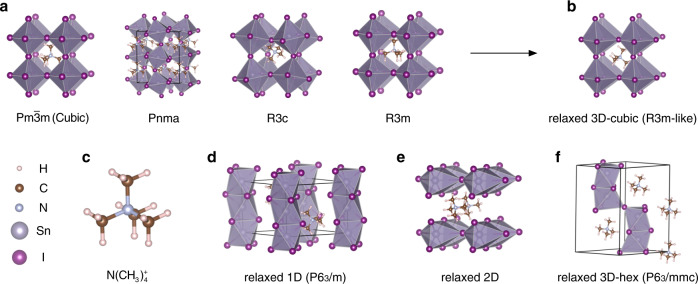
The initial and relaxed structures of N(CH_3_)_4_SnI_3_. **a** The initial 3D-cubic structures with symmetry *Pm*$\bar 3$*m*, *Pnma*, *R3c*, and *R3m*. **b** The relaxed 3D-cubic structure from the initial ones in **a**. **c** The organic molecule N(CH_3_)_4_^+^ located at A site. **d** The relaxed 1D (*P6*_*3*_*/m*) structure which represents the experimental structure at the ambient pressure. **e** The relaxed 2D structure and **f** the relaxed 3D-hex (*P6*_*3*_*/mmc*) structure.

In order to predict the stable crystal structure of N(CH_3_)_4_SnI_3_, seven different structures are considered in the present study, including five three-dimensional (3D) structures as shown in Fig. 1a, f, one two-dimensional (2D) structure in Fig. 1e and one one-dimensional (1D) structure in Fig. 1d.

Four 3D structures in Fig. 1a are derived by replacing the A-site cation of inorganic ABX_3_ perovskite by the non-polar N(CH_3_)_4_^+^ group and B-site cation by Sn^2+^. It is well known that the perovskite ABX_3_ compounds can have different lattice symmetries at different temperatures, e.g., the cubic structure with the *Pm*$\bar 3$*m* space group at high temperatures and the structures with the *Pnma*, *R3c*, and *R3m* space groups at lower temperatures, which can be considered as the distorted cubic structures with the tilting or rotating of the BX_6_ octahedrons and the displacement of the B cation. The four structures with *Pm*$\bar 3$*m*, *Pnma*, *R3c,* and *R3m* space groups are all adopted as the initial N(CH_3_)_4_SnI_3_ structures in the present study and denoted as 3D-cubic structures. For the ABX_3_ perovskites with large A cations (e.g., BaMnO_3_), the hexagonal structure with the space group *P6*_*3*_*/mmc* had also been reported, so the *P6*_*3*_*/mmc* structure is also adopted as an initial structure of N(CH_3_)_4_SnI_3_ and denoted as 3D-hex in Fig. 1f.

In the experiments, Liu et al. synthesized N(CH_3_)_4_PbI_3_ at room temperature and ambient pressure^15^. The characterization showed that it has the 1D (*P6*_*3*_*/m*) structure, where the PbI_6_ octahedrons share three I atoms with adjacent ones and form lines in the lattice. Because of the similarity of Pb and Sn, we also consider the 1D structure as the initial N(CH_3_)_4_SnI_3_ structure (see Fig. 1d). Furthermore, we construct the 2D structure (see Fig. 1e) as the initial structure, where SnI_6_ octahedrons share two I atoms with adjacent ones and then form a 2D plane. Although 1D, 2D, and 3D-hex are not perovskite structures, we still consider them as N(CH_3_)_4_SnI_3_ initial structures in order to check the stability of the 3D-cubic structures.

In order to predict the stability of the seven structures under different pressures, the total enthalpy $H_{{\mathrm{tot}}}$ is calculated as

1 $$\begin{array}{*{20}{c}} {H_{{\mathrm{tot}}} = U + PV} \end{array},$$

where *U* is the internal energy of the supercell, *P* is the hydrostatic pressure, and *V* is the volume of the supercell. The total enthalpy per formula unit ($H_{{\mathrm{tot}}}$/f.u.) of the structures relaxed from *Pm*$\bar 3$*m*, *Pnma*, *R3c,* and *R3m* are in the range of −99.406 to −99.394 eV. The absolute difference of $H_{{\mathrm{tot}}}$/f.u. is less than 12 meV, i.e., 0.57 meV/atom. All the $H_{{\mathrm{tot}}}$/f.u. of four initial 3D-cubic structures are listed in Supplementary Table 1. The difference is negligible and can be attributed to the numerical error and the different orientations of the N(CH_3_)_4_^+^ at A site. According to the symmetry analysis^16^, we found that the symmetries of the relaxed structures are all *R3m*-like (see Fig. 1b), i.e., whatever the initial structure is (*Pm*$\bar 3$*m*, *Pnma*, *R3c*, or *R3m*), the relaxed structures are the same. We use the average value of $H_{{\mathrm{tot}}}$/f.u. = −99.402 eV for the 3D-cubic relaxed structure hereafter (see Table 1). The detailed 3D-cubic crystal structure and lattice parameters are shown in Supplementary Fig. 1 and Supplementary Table 2. In the relaxed structure, the SnI_6_ octahedrons sit in the corners of the cubic box. There is no tilt of SnI_6_ octahedrons, but the Sn atom displaces from the center of the SnI_6_ octahedron, producing three short Sn–I bonds and three longer Sn–I bonds.

**Table 1:** Total enthalpy per formula unit $H_{{\mathrm{tot}}}$/f.u. (in eV) of the relaxed structures under 0 and 6 GPa.

	Pressure	3D-cubic	2D	1D	3D-hex
$H_{{\mathrm{tot}}}$/f.u.	0 GPa	−99.402	−99.265	−99.514	−99.434
	6 GPa	−89.008	−88.348	−88.956	−88.923

Figure 1c shows the organic molecule N(CH_3_)_4_^+^ at A site. The different directions of the N–C bonds in N(CH_3_)_4_^+^ can lead to different initial structures despite the same lattice space group of ABX_3_. However, we found that the difference of the total enthalpies is small, so the effect of the initial rotation of N(CH_3_)_4_^+^ on the relaxed structures can be neglected. More details can be found in Supplementary Table 3.

The relaxed 1D and 2D structures are shown in Figs. 1d, e. $H_{{\mathrm{tot}}}$/f.u. are −99.514 and −99.265 eV for 1D and 2D structures, respectively (see Table 1). Under 0 GPa, the energy of 1D structure is about 100 meV lower than that of 3D-cubic and 250 meV lower than that of 2D, which means the 1D structure is more stable under the ambient pressure. Our calculation is consistent with the experimental result^15^.

Figure 1f shows the relaxed 3D-hex structure. $H_{{\mathrm{tot}}}$/f.u. under 0 GPa is about −99.434 eV (see Table 1) and it is about 30 meV lower than that of 3D-cubic structure but still about 80 meV higher than that of 1D structure, meaning that the 3D-hex structure is also more stable than 3D-cubic structure under the ambient pressure.

**Fig. 2:**
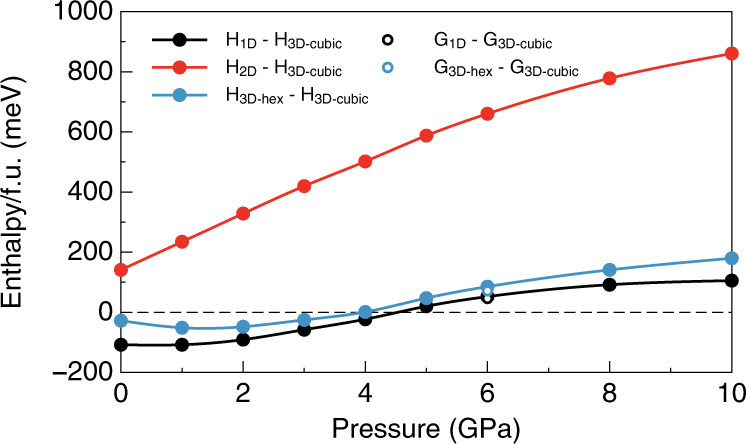
Enthalpy differences as functions of hydrostatic pressure. Solid black circles: enthalpy differences between 1D and 3D-cubic structures of N(CH_3_)_4_SnI_3_. Solid red circles: enthalpy differences between 2D and 3D-cubic structures. Solid blue circles: enthalpy differences between 3D-hex and 3D-cubic structures. The calculated Gibbs free energy differences between 3D-cubic and 1D (open black circle), 3D-hex (open blue circle) structures at 6 GPa and 300 K are also marked.

Although the 3D-cubic perovskite structure is not the most energetically preferred at 0 GPa, it was suggested that the 3D-cubic perovskite can be synthesized and the electronic structure can be modified under a high pressure^17^. So, we apply a hydrostatic pressure up to 10 GPa on the 1D, 2D, 3D-cubic, and 3D-hex structures, respectively, to investigate the behavior of the structure under different pressures. We compare $H_{{\mathrm{tot}}}$/f.u. differences between 3D-cubic and 2D $\left( {H_{{\mathrm{2D}}} - H_{{\mathrm{3D \hbox{-} cubic}}}} \right)$, 1D $\left( {H_{{\mathrm{1D}}} - H_{{\mathrm{3D \hbox{-} cubic}}}} \right)$, and 3D-hex $\left( {H_{{\mathrm{3D \hbox{-} hex}}} - H_{{\mathrm{3D \hbox{-} cubic}}}} \right)$ structures in Fig. 2. Under pressures below 4.5 GPa, H_1D_ − H_3D-cubic_ < 0, which means the 1D structure is more stable than 3D-cubic structure (see black line in Fig. 2). When the pressure increases above 4.5 GPa, $H_{{\mathrm{1D}}} - H_{{\mathrm{3D \hbox{-} cubic}}} \,> \, 0$, where the 3D-cubic structure becomes more energetically preferred than 1D. Similarly, when the pressure is higher than 4.5 GPa, the 3D-cubic structure is more stable than 3D-hex structure (see blue line in Fig. 2). Meanwhile, since $H_{{\mathrm{2D}}} - H_{{\mathrm{3D \hbox{-} cubic}}}\, > \, 0$ in the whole pressure range, the 3D-cubic structures are always more stable than 2D ones (see red line in Fig. 2). Our calculation indicates that the 3D-cubic N(CH_3_)_4_SnI_3_ (with the *R3m* space group) is more stable under high pressure, which makes the synthesis of 3D-cubic N(CH_3_)_4_SnI_3_ perovskite possible.

We also considered the vibration contribution to the stability of different phases through calculating the Gibbs free energy $G$ as following^18^:

2 $$G = \, H_{{\mathrm{tot}}} + F_{{\mathrm{vib}}}\left( T \right) \\ = \, H_{{\mathrm{tot}}} + \frac{1}{2}\mathop {\sum }\limits_{i,{\mathbf{q}}} \hbar \omega _{i,{\mathbf{q}}} + k_{\mathrm{B}}T\mathop {\sum }\limits_{i,{\mathbf{q}}} \ln \left[ {1 - {\mathrm{exp}}\left( { - \frac{{\hbar \omega _{i,{\mathbf{q}}}}}{{k_{\mathrm{B}}T}}} \right)} \right]$$

where $F_{{\mathrm{vib}}}(T)$ is the vibration contribution (including the internal vibrational energy and vibrational entropy) to the Gibbs free energy, $\omega _{i,{\boldsymbol{q}}}$ is the phonon frequency of band index *i* and wave vector **q**, *T* is the temperature, *k*_B_ is the Boltzmann constant, $\hbar$ is the reduced Planck’s constant. As shown in Fig. 2, at 6 GPa and *T* = 300 K, the calculated $G$ difference (per formula unit) between 3D-cubic and 1D NH_4_SnI_3_ is 46 meV, which is close to the enthalpy difference (52 meV); the calculated $G$ difference between 3D-cubic and 3D-hex NH_4_SnI_3_ is 71 meV, which is also close to the enthalpy difference (85 meV). Obviously, the vibration contribution to the $G$ differences is only a few meV, much smaller than the large positive value of the $H_{{\mathrm{tot}}}$ difference at 6 GPa. Therefore, the 3D-cubic structure is still more stable than 3D-hex and 1D structure, and our conclusion about the stability of different phases is not changed after the vibration contribution is considered.

Since the molecule N(CH_3_)_4_^+^ is at A site, we also considered the van der Waals (vdW) interactions for the structural relaxation. When the vdW interaction is included in the relaxation, the lattice constant is about 6.73 Å, which is smaller than ~7.04 Å (without vdW). However, for the pressure-dependent total enthalpy ($H_{{\mathrm{tot}}}$/f.u.) which is crucial to determine the stable state at high pressure, the conclusion is not changed, i.e., the 3D-cubic is the most stable structure at high pressure. The detailed results can be found in Supplementary Fig. 2.

Since N(CH_3_)_4_SnI_3_ can phase-separate into N(CH_3_)_4_I and SnI_2_ and then become unstable, we also calculated the formation enthalpy (energy cost) of the chemical reaction N(CH_3_)_4_I  + SnI_2_ → N(CH_3_)_4_SnI_3_ in the pressure range of 0–6 GPa. The calculated energy costs for 3D-cubic N(CH_3_)_4_SnI_3_ are all negative (see Supplementary Fig. 3), which means that 3D-cubic N(CH_3_)_4_SnI_3_ is thermodynamically stable with respect to the phase-separation, and furthermore, it may be synthesized through mixing N(CH_3_)_4_I and SnI_2_ at high pressure.

### Dynamic stability of ferroelectric structure

**Fig. 3:**
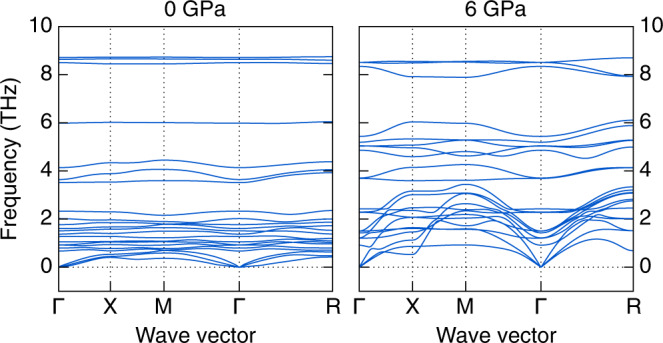
Phonon dispersion of *R3m* N(CH_3_)_4_SnI_3_ under 0 and 6 GPa. The high-symmetry wave vectors are Γ (0, 0, 0), X (0, 1/2, 0), M (1/2, 1/2, 0), and R (1/2, 1/2, 1/2).

In order to investigate the dynamic stability of the *R3m* structure, we calculated its phonon dispersion under 0 and 6 GPa via the phonopy package^19^. The results are shown in Fig. 3. Both the phonon dispersions at 0 and 6 GPa have no imaginary frequency, which indicates that the 3D-cubic structure is dynamically stable under both ambient and high pressures. The phonon dispersion calculated at 0 GPa is similar to that of 6 GPa, so we can conclude that even after the pressure is released, the 3D-cubic structure can still remain stable.

### Spontaneous polarization

In order to predict whether 3D-cubic N(CH_3_)_4_SnI_3_ (see Fig. 1b) has a large spontaneous polarization, we calculated the spontaneous polarization of N(CH_3_)_4_SnI_3_ by using the Berry phase method^20^. The overall spontaneous polarization ${\mathbf{P}}$ of the ferroelectric phase can be written as

3 $$\begin{array}{*{20}{c}} {{\mathbf{P}} = \left( {P_x,P_y,P_z} \right) = {\mathbf{P}}_{{\mathrm{ferro}}} - {\mathbf{P}}_{{\mathrm{para}}}} \end{array}$$

where ${\mathbf{P}}_{{\mathrm{ferro}}}$ $\left( {{\mathbf{P}}_{{\mathrm{para}}}} \right)$ is the polarization of the ferroelectric (paraelectric) phase. *P*_*x*_, *P*_*y*_, *P*_*z*_ are the three components of ${\mathbf{P}}$ along [100], [010], and [001] directions, respectively.

**Fig. 4:**
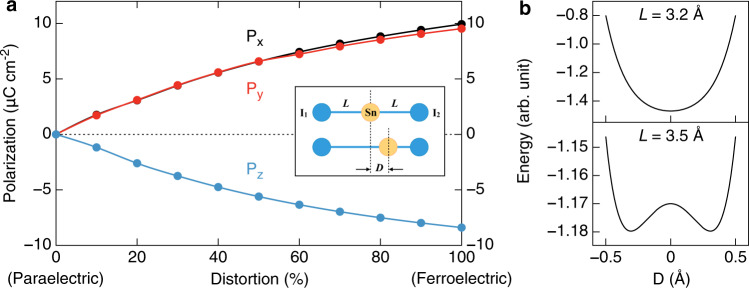
Spontaneous polarization and the I–Sn–I model for explaining the ferroelectricity mechanism. **a** Polarization of the 3D-cubic *R3m* N(CH_3_)_4_SnI_3_ as a function of the atomic distortion from the paraelectric to the ferroelectric phase. The black, red, and blue lines indicate *P*_*x*_, *P*_*y*_, and *P*_*z*_ component of polarization, respectively. Inset: illustration of the linear I–Sn–I model. **b** The total energy as a function of the Sn^2+^ displacement for *L* = 3.2 and 3.5 Å, respectively.

The calculated polarization of the ferroelectric phase (*R3m*) of N(CH_3_)_4_SnI_3_ is shown in Fig. 4a. In the calculation of ferroelectric polarization, the tetragonal structure with $P\bar 4m2$ space group is used as the paraelectric phase. All the atoms are moved manually from the paraelectric phase to the ferroelectric phase to form the intermediate phases. The atomic displacements are indicated by the distortion percentage (the horizontal axis of Fig. 4a).

With respect to the paraelectric phase, the overall spontaneous polarization is found to be ${\mathbf{P}} = \left( {9.94,\,9.53,\, -8.40} \right)\,\upmu {\mathrm{C}}\;{\mathrm{cm}}^{ - 2}$. The polarization is nearly along the $[11\bar 1]$ direction with a magnitude of $\left| {\mathbf{P}} \right| = 16.13\,\upmu {\mathrm{C}}\;{\mathrm{cm}}^{ - 2}$. In order to examine the contribution of the N(CH_3_)_4_^+^ molecule displacement to ${\mathbf{P}}$, we fix Sn and I positions and move N(CH_3_)_4_^+^ manually from the paraelectric phase to the ferroelectric phase. The contribution of the N(CH_3_)_4_^+^ off-centering displacement is ${\mathbf{P}}_{{\mathrm{org}}} = \left( { - 0.49,\, - 0.53,\, - 0.33} \right)\;\upmu {\mathrm{C}}\;{\mathrm{cm}}^{ - 2}$, which is nearly along $[\bar 1\bar 1\bar 1]$ with the magnitude of $0.79\,\upmu{\mathrm{C}}\;{\mathrm{cm}}^{ - 2}$. In contrast, by fixing N(CH_3_)_4_^+^ but relaxing the positions of Sn and I atoms, the calculated contribution of Sn–I off-centering displacement is ${\mathbf{P}}_{{\mathrm{inorg}}} = \left( {10.36,\,10.27,\, -8.03} \right)\;\upmu {\mathrm{C}}\;{\mathrm{cm}}^{ - 2}$, with the magnitude of $16.65\,\upmu {\mathrm{C}}\;{\mathrm{cm}}^{ - 2}$ pointing to $[11\bar 1]$, which is one order of magnitude larger than ${\mathbf{P}}_{{\mathrm{org}}}$. Therefore, we can conclude that the spontaneous polarization ${\mathbf{P}}$ is mainly attributed to the Sn–I displacement.

Moreover, because of the non-polar feature of N(CH_3_)_4_^+^ group, we can further ignore the effect originating from the internal polarization of N(CH_3_)_4_^+^ molecule. As a comparison, the available measured polarization of CH_3_NH_3_PbI_3_ is about $7\;\upmu {\mathrm{C}}\;{\mathrm{cm}}^{ - 2}$ (ref. ^11^). So the polarization of N(CH_3_)_4_SnI_3_ is large enough and therefore can play a positive role in suppressing the recombination of photon-generated electron-hole pairs in the PV devices.

### Band structure and band gap

After making sure that the *R3m* N(CH_3_)_4_SnI_3_ has strong ferroelectricity and can also be stable, now we will study its band structure and band gap (*E*_g_) in order to ensure that it is suitable as a light-absorber semiconductor in solar cells.

The band structure and band gap are calculated using the generalized gradient approximation (GGA) with the exchange-correlation functional in the Perdew–Burke–Ernzerhof (PBE) form, and the spin-orbit coupling (SOC) effect is also considered. For 1D N(CH_3_)_4_SnI_3_ structure, the experimentally measured band gap is 2.6 eV^21^ which is used as the reference for us to choose the appropriate functional for predicting the properties of N(CH_3_)_4_SnI_3_. We have tried four different methods, including PBE, PBE+SOC, HSE06, and HSE06+SOC, to calculate the 1D N(CH_3_)_4_SnI_3_ band gap and the results are 2.76, 2.64, 3.47, and 3.33 eV (the corresponding band structure and density of states (DOS) are shown in Supplementary Fig. 4), respectively. Obviously, the calculated band gap of 1D N(CH_3_)_4_SnI_3_ using PBE+SOC agrees well with the experimental data, indicating that the PBE+SOC method may be more suitable for predicting the band gap of N(CH_3_)_4_SnI_3_ accurately. Therefore, we choose PBE+SOC to calculate the band structure and band gap for 3D-cubic N(CH_3_)_4_SnI_3_.

**Fig. 5:**
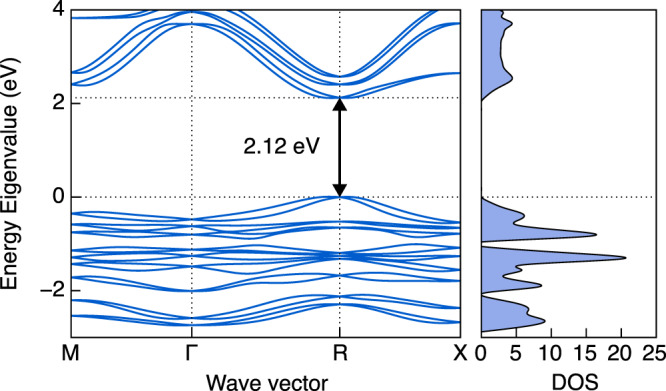
Band structure and DOS of *R3m* N(CH_3_)_4_SnI_3_. The high-symmetry wave vectors are M(1/2, 1/2, 0), Γ(0, 0, 0), R(1/2, 1/2, 1/2), and X(0, 1/2, 0). The Fermi level is shifted to 0 eV.

The calculated band structure and DOS of the *R3m* 3D-cubic N(CH_3_)_4_SnI_3_ performed by PBE+SOC under 0 GPa are shown in Fig. 5. We can see that the *R3m* 3D-cubic N(CH_3_)_4_SnI_3_ is a direct band gap semiconductor at ambient condition, where the valence band maximum (VBM) and the conduction band minimum (CBM) are both located at the R point. The optical band gap *E*_g_ = 2.12 eV.

**Fig. 6:**
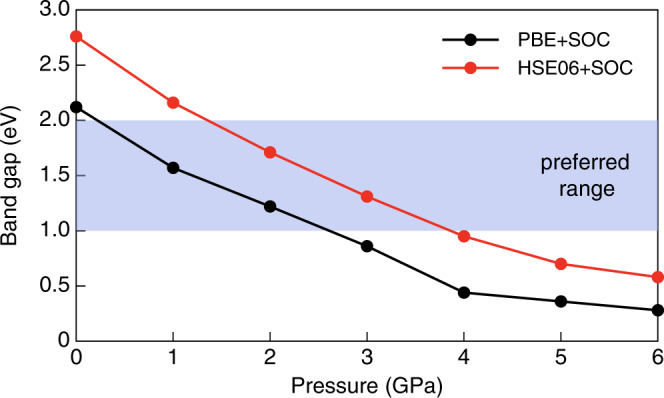
Band gap of *R3m* 3D-cubic N(CH_3_)_4_SnI_3_ as a function of external hydrostatic pressure. Black line: calculated using PBE+SOC, red line: calculated using HSE06+SOC. The blue color shows the band gap range suitable for the light-absorber semiconductor in solar cells.

The band gap of N(CH_3_)_4_SnI_3_ can be tuned effectively through external pressure. In Fig. 6, we plot the calculated *E*_g_ using both PBE+SOC and HSE06+SOC under hydrostatic pressures up to 6 GPa. For the PBE+SOC case, as the pressure increases from 0 to 6 GPa, *E*_g_ decreases from 2.12 to 0.28 eV. For the HSE06+SOC case, *E*_g_ decreases from 2.76 to 0.58 eV with pressure increasing from 0 to 6 GPa. Although the band gaps calculated by the two methods have different absolute values, they have the same trend with respect to the pressure, i.e., with the increasing pressure, the band gaps both decrease with a similar slope. The monotonic decrease of the band gap with the hydrostatic pressure can be understood according to the composition of the VBM and CBM states. For *R3m* N(CH_3_)_4_SnI_3_, the VBM state is mainly composed of anti-bonding hybridization between Sn *s* and I *p* orbitals, and the CBM state is mainly the non-bonding Sn *p* state^22^. When the pressure increases, the lattice constant decreases, so the Sn–I bond length is shortened, which increases the *s*–*p* hybridization between Sn and I and shifts the VBM level up. In contrast, the Sn *p* states is weakly affected under pressure, so the CBM level remains unchanged. As a result, the band gap decreases monotonically as the pressure increases.

Band structure engineering has been implemented to increase the PCE of the metal-halide perovskite solar cells by reducing the optical band gap to 1.3–1.4 eV^17^. The thin-film materials with *E*_g_ = 1.0–2.0 eV are proven to be suitable for light-absorber semiconductors in solar cells^7,8^, which is shown by the blue area in Fig. 6. In our calculation, when the pressure increases to 0.25 GPa, the band gap decreases to 2.0 eV. When the external pressure increases to 2.6 GPa, the band gap decreases to 1.0 eV. These results suggest that after the sample is synthesized under high pressure, the subsequent pressure release process can be controlled to tune the electronic band gap to fit the requirement of the light-absorber semiconductor in solar cells.

In order to demonstrate the potential application of the *R3m* N(CH_3_)_4_SnI_3_, we also calculated the optical absorption spectrum for *R3m* N(CH_3_)_4_SnI_3_. The results are shown in Supplementary Fig. 5. The calculated absorption coefficient increases sharply as the photon energy is above the band gap, so N(CH_3_)_4_SnI_3_ can absorb visible light effectively and act as a potential high-efficiency light-absorber semiconductor in solar cells.

## Discussion

Understanding the origin of the ferroelectric feature of 3D-cubic N(CH_3_)_4_SnI_3_ can help the design of the ferroelectric OIHPs. The symmetry of the ABX_3_ perovskite structure can be estimated by the empirical Goldschmit tolerance factor $t = \frac{{r_{\mathrm{A}} + r_{\mathrm{B}}}}{{\sqrt 2 \left( {r_{\mathrm{B}} + r_{\mathrm{X}}} \right)}}$, where $r_{\mathrm{A}}$, $r_{\mathrm{B}}$ and $r_{\mathrm{X}}$ are the radius of A, B and X, respectively. The structure tends to be *Pnma* when *t* < 0.8, $Pm\bar 3m$ (cubic) when 0.8 < *t* < 1.0, and *R3c* or *R3m* when *t* > 1.0 (ref. ^15^). The radius of A-site group can determine the value of *t* and then play an important role in the structure symmetry. In N(CH_3_)_4_SnI_3_, the ionic radius of N(CH_3_)_4_^+^ is about 3.2 Å and the corresponding *t* = 1.13 (see Table 2). The relatively large N(CH_3_)_4_^+^ makes the BX_6_ octahedrons untilted. Meanwhile, the Sn^2+^ displaces from the center of SnI_6_ octahedron in order to form a better bonding with the neighboring I^−^. The overall structure is *R3m*-like. It appears that the large tolerance factor is crucial to the ferroelectricity in 3D-cubic N(CH_3_)_4_SnI_3_.

**Table 2:** Ionic radius of N(CH_3_)_4_^+^, NH_4_^+^, Cs^+^, Sn^2+^, I^−^ and the tolerance factor *t* of ASnI_3_ (*A* = N(CH_3_)_4_^+^, NH_4_^+^, and Cs^+^).

*A*	Ionic radius (Å)	Ref.	*t* (ASnI_3_)
N(CH_3_)_4_^+^	3.20	^15^	1.13
NH_4_^+^	1.61	^30^	0.80
Cs^+^	1.78	^30^	0.83
Sn^2+^	1.18	^31^	–
I^−^	2.20	^31^	–

We double check this conclusion by investigating NH_4_SnI_3_ and CsSnI_3_, in which the A-site groups are smaller. The radii of NH_4_^+^ and Cs^+^ are 1.61 and 1.78 Å (see Table 2), respectively. The tolerance factors are both relatively smaller (0.80 and 0.83, respectively, see Table 2). We construct the initial structures of NH_4_SnI_3_ and CsSnI_3_ from $Pm\bar 3m$, *Pnma*, *R3c*, and *R3m* and relax all the structures. The calculated total energies of the relaxed structures are listed in Table 3. The most stable structures of both NH_4_SnI_3_ and CsSnI_3_ are *Pnma*, which means the ferroelectricity is suppressed. The previous experimental and theoretical results also gave the same results^23^. Furthermore, we investigate the role of A group on the ground state structure by increasing the volume of $Pm\bar 3m$, *Pnma*, *R3c*, *R3m* CsSnI_3_ and fixing the volume during the relaxation. We find that *R3m* CsSnI_3_ structure tends to be the most stable at large volume (see Supplementary Fig. 6). Since the increasing of the volume can be regarded as equivalent to the enlargement of the A-site group and the increasing of *t*, so the results indicated that the large *t* makes N(CH_3_)_4_SnI_3_ form the ferroelectric structure.

**Table 3:** The total energy/f.u. (in eV) of NH_4_SnI_3_ and CsSnI_3_ relaxed from initial structures ${{Pm}}\bar 3{{m}}$, *Pnma*, *R3c,* and *R3m*.

	$Pm\bar 3m$	*Pnma*	*R3c*	*R3m*
NH_4_SnI_3_	−34.376	−34.631	−34.601	−34.463
CsSnI_3_	−14.041	−14.089	−14.068	−14.040

The fact that a perovskite with a large tolerance factor *t* tends to be ferroelectric can be understood as follows. When the Sn–I distances in the cubic paraelectric structure is large (a large *t*), the second-order Jahn–Teller effect (electronic hybridization) is more important, which results in the off-centering Sn^2+^ displacements and thus ferroelectric polarization. For small *t*, the Sn–I bond lengths are small so that the Pauli repulsion between the Sn^2+^ ion and I^−^ ion is strong, which keeps the Sn ions at the center of the octahedrons. Note that the same mechanism also explains the ferroelectricity in the classic inorganic perovskite ferroelectric: BaTiO_3_ with a large *t* is ferroelectric while SrTiO_3_ with a small *t* is not.

To see the competition between the electronic hybridization and Pauli repulsion clearly, let us consider a simple linear I–Sn–I model (see the inset of Fig. 4a). We adopt the repulsion part of the Lennard–Jones potential to approximate the total Pauli repulsion energy: $E_{{\mathrm{rep}}} = \frac{a}{{\left( {L + D} \right)^{12}}} + \frac{a}{{\left( {L - D} \right)^{12}}}$ (*a* > 0), where *L* is original Sn–I_1_ and Sn–I_2_ bond distance and *D* is the displacement of the Sn^2+^. With a simple tight-binding model, the energy lowering due to the hybridization between the empty orbital of Sn^2+^ ion and occupied orbital of the I^−^ ion (for simplicity, we consider only one orbital for each ion): $E_{{\mathrm{hop}}} \approx - \frac{{\left( {t_{{\mathrm{Sn - }}I_1}} \right)^2}}{\Delta } - \frac{{(t_{{\mathrm{Sn - }}I_2})^2}}{\Delta }$, where $t_{{\mathrm{Sn - }}I_1}$ $\left( {t_{{\mathrm{Sn - }}I_2}} \right)$ is the hopping integral between Sn and I_1_(I_2_) ion, $\Delta$(>0) is the energy difference between the empty orbital of Sn^2+^ and occupied orbital of the I^−^. According to Harrison’s method^24^, the dependence of the hopping integral on the bond distance can be written as: $t_{{\mathrm{Sn - }}I_1} = \frac{b}{{\left( {L + D} \right)^2}}$ and $t_{{\mathrm{Sn - }}I_2} = \frac{b}{{\left( {L - D} \right)^2}}$ (*b* is a constant). In Fig. 4b, we plot the total energy $E_{{\mathrm{tot}}} = E_{{\mathrm{rep}}} + E_{{\mathrm{hop}}}$ as a function of *D* for two cases with different Sn–I_1_(I_2_) bond distance (*L* = 3.2 or 3.5 Å) in the centrosymmetric linear model. As can be seen in Fig. 4b, in the case of short *L* (i.e., 3.2 Å), the energy minimum locates at *D* = 0, i.e., there is no ferroelectric instability. When the *L* is large (i.e., 3.5 Å, which corresponds to the 3D-cubic structure in our work), the energy curve becomes a double-well potential, indicating a ferroelectric instability (see Fig. 4b). In summary of above discussion, the Pauli repulsion dominates when the Sn–I_1_(I_2_) distance is small, while the second-order Jahn–Teller effect dominates in the case of long Sn–I_1_(I_2_) distance.

To summarize, the Pb-free hybrid perovskite N(CH_3_)_4_SnI_3_ with the non-polar molecular cation N(CH_3_)_4_^+^ at A site and *R3m* structure is found to be a photovoltaic light-absorber semiconductor with robust and strong ferroelectricity. Its calculated spontaneous polarization is as large as 16.13 μC cm^−2^ along $[11\bar 1]$ direction. In contrast to the transient ferroelectricity of CH_3_NH_3_PbI_3_ which is sensitive to the orientation ordering of the polar molecular cation, the large intrinsic polarization of N(CH_3_)_4_SnI_3_ results mainly from the Sn off-centering displacement induced by the large size of the N(CH_3_)_4_^+^ molecular cation and large structural tolerance factor, so it is permanent and robust at room temperature. The ferroelectric *R3m* structure is the ground state under hydrostatic pressure higher than 3 GPa, and is dynamically stable under the 0–6 GPa pressure according to the calculated phonon dispersion. Therefore, we propose that the ferroelectric *R3m* phase of N(CH_3_)_4_SnI_3_ can be synthesized under high pressure (3 GPa) and can remain stable under ambient condition after the pressure is released. Its band gap can be tuned effectively from 2.12 to 1.0 eV by applying a hydrostatic pressure in the range of 0–3 GPa and its absorption coefficient of visible light is high. Therefore, this Pb-free and ferroelectric hybrid perovskite N(CH_3_)_4_SnI_3_ is a suitable light-absorber semiconductor for solar cells. Its spontaneous polarization can suppress the recombination of the photo-generated electron–hole pairs, so it can be a high-efficiency light-absorber semiconductor in solar cells.

Unlike the traditional solar cells based on the heterogeneous p–n junction, the OIHPs semiconductor N(CH_3_)_4_SnI_3_ with strong ferroelectricity and suitable band gap may have large bulk photovoltaic effect and can be used for designing homogeneous bulk photovoltaic devices. Our work paves a way for designing high-efficiency and Pb-free OIHPs solar cells.

## Methods

The structural relaxation and electronic structure calculations are performed using the plane-wave pseudopotential method based on the density functional theory (DFT) as implemented in the Vienna Ab initio Simulation Package (VASP)^25^. For the exchange-correlation functional, we tested both the semi-local GGA in the PBE^26^ form and the Heyd–Scuseria–Ernzerhof (HSE)^27^ hybrid functional both with the SOC included. For the plane-wave basis, we use an energy cutoff *E*_cut_ = 520 eV. In the relaxation process, the ionic relaxation loop breaks when the change of energy reaches 1.0 × 10^−6^ eV and forces are below 0.005 eV/Å. A 5 × 5 × 5 Monkhorst–Pack k-point mesh centered at Γ point is used for structure relaxation, dynamic stability, ferroelectric calculations. We involve 5 valence electrons for N atom, 4 for C atom, 1 for H atom, 14 for Sn atom, and 7 for I atom. We adopted the DFT-D3 correction method with Becke–Jonson damping^28^ as implemented in VASP to include the vdW effect.

To obtain the phonon dispersion, we first calculate the force constants using the finite displacement method. Then, phonon frequencies and eigenvectors are calculated with the phonopy software package^19^.

The optical absorption spectra and spontaneous polarization are calculated based on the converged charge density of the static electronic structure calculations. The imaginary part of the frequency-dependent dielectric function is calculated using the random phase approximation as formalized by Gajdoš et al.^29^, and the real part is derived using the Kramers–Kronig transformation. Then, the frequency-dependent optical absorption coefficient is obtained from the dielectric function. The spontaneous ferroelectric polarization is calculated using the Berry phase method^20^.

### Reporting summary

Further information on research design is available in the Nature Research Reporting Summary linked to this article.

## Supplementary information

Supplementary Information

Reporting Summary

## Data Availability

The data that support the findings of this study are available from the corresponding author upon reasonable request.
