# Supplementary material for: Lead-free hybrid perovskite N(CH3)4SnI3 with robust ferroelectricity induced by large and non-polar N(CH3)4+ molecular cation
Source: Nat Commun. 2021 Jan 27;12:637. doi: 10.1038/s41467-021-20889-y (PMC7840759; doi:10.1038/s41467-021-20889-y)
Supplement: Supplementary file 1 — Supplementary Information [file 41467_2021_20889_MOESM1_ESM.pdf]

Supplementary Information for

Lead-free Hybrid Perovskite $N(CH_3)_4SnI_3$ with Robust Ferroelectricity Induced by Large and Non-Polar $N(CH_3)_4^+$ Molecular Cation

Hai Wei[1,2], Yali Yang[1,2], Shiyou Chen[3,*] and H. J. Xiang[1,2,†]

[1] Key Laboratory of Computational Physical Sciences (Ministry of Education), State Key Laboratory of Surface Physics, and Department of Physics, Fudan University, Shanghai 200433, China

[2] Collaborative Innovation Center of Advanced Microstructures, Nanjing 210093, China

[3] Key Laboratory of Polar Materials and Devices (Ministry of Education), East China Normal University, Shanghai 200241, China

* chensy@ee.ecnu.edu.cn † hxiang@fudan.edu.cn

Supplementary Note 1. Construction of $Pm\overline{3}m$, *Pnma*, *R3c* and *R3m* $N(CH_3)_4SnI_3$ structures and the relaxed 3D structure

For the 3D ABX_3 perovskite materials, BX_6 octahedrons are located at the corners of the cubic cell, and the A atom is embedded at the center. We firstly construct this $ASnI_3$ structure with the $Pm\overline{3}m$, *Pnma*, *R3c* and *R3m* space groups. Then we replace the A site by the organic group $N(CH_3)_4^+$. The whole supercell system is relaxed completely to obtain the lowest-energy structure. The total enthalpy H_{tot} of the four initial 3D-cubic structures are listed in the Supplementary Table 1.

The relaxed 3D-cubic $N(CH_3)_4SnI_3$ structure is shown in Supplementary Fig. 1 and the geometric crystal structural parameters are listed in Supplementary Table 2. In 3D-cubic $N(CH_3)_4SnI_3$, SnI_6 octahedrons are untilted. The Sn^{2+} ions displace from the center, so the bond angle ∠Sn-I1-Sn = ∠I1-Sn-I4 = 160.2°. Because of the Sn^{2+} ions' displacements, the Sn-I1, Sn-I2 and Sn-I3 bonds are longer (>4.2 Å). The other bonds Sn-I4, Sn-I5 and Sn-I6 are shorter (around 2.9 Å).

Supplementary Table 1 The total enthalpy per formula unit H_{tot}/f.u. (in eV) of the 3D-cubic $N(CH_3)_4SnI_3$ relaxed from the initial structures with the space groups $Pm\overline{3}m$, *Pnma*, *R3c* and *R3m* at 0 GPa and 6 GPa.

		$Pm\overline{3}m$	*Pnma*	*R3c*	*R3m*
H_{tot}/f.u.	0 GPa	-99.406	-99.405	-99.394	-99.403
	6 GPa	-89.008	-89.002	-88.988	-89.008

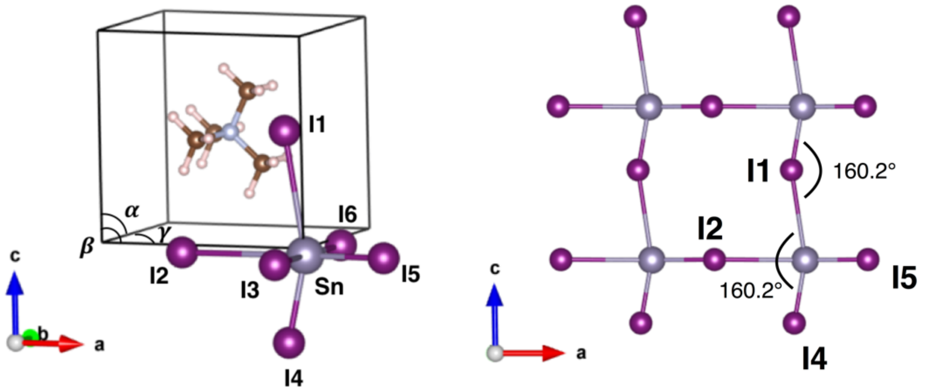

Supplementary Fig. 1 The relaxed 3D-cubic $N(CH_3)_4SnI_3$ structure. Only one BX_6 octahedron is shown in the corner.

Supplementary Table 2 The lattice constants and Sn-I bond lengths of 3D-cubic $N(CH_3)_4SnI_3$ structure. All lengths are in Å.

Lattice Constant	a	b	c	α	β	γ
	7.05	7.24	7.05	91.4°	90.1°	91.0°
Bond Length	Sn-I1	Sn-I2	Sn-I3	Sn-I4	Sn-I5	Sn-I6
	4.20	4.20	4.34	2.95	2.91	2.93

Supplementary Note 2. Effect of the direction of $N(CH_3)_4^+$ on the total energy

When constructing the initial structures for structural relaxation, there are many possible directions of the organic group $N(CH_3)_4^+$ in the 3D $N(CH_3)_4SnI_3$. In order to investigate the influence of the initial direction of the organic group on the relaxed structures, we constructed 5 *Pnma* initial structures with different directions of $N(CH_3)_4^+$ at A site and then relaxed these structures. Supplementary Table 3 shows the relaxed total enthalpy per formula unit (H_{tot}/f.u.) of these structures at 0 GPa. The total energy differences of the relaxed structures from the 5 initial structures are within 4 meV per formula unit, which is about 0.19 meV/atom. The difference of the total energy is small enough so the effect of the rotation of $N(CH_3)_4^+$ on the relaxed structures can be neglected. The relaxed structures are also compared and no obvious difference can be found, indicating that the initial directions of $N(CH_3)_4^+$ do not influence the relaxed structure.

Supplementary Table 3 The H_{tot}/f.u. *Pnma* $N(CH_3)_4SnI_3$ relaxed from 5 initial structures with different organic molecule directions at A site.

	1	2	3	4	5
H_{tot}/f.u. (eV)	-99.406	-99.407	-99.409	-99.406	-99.405

Supplementary Note 3. Effect of the vdW interaction on the structural relaxation and the total energy

Since it is a molecule $N(CH_3)_4^+$ at A site, we considered the vdW effect on the structural relaxation. The lattice constant is ~6.73 Å (PBE+vdW), which is a little smaller than ~7.04 Å (PBE). However, for the total enthalpy (H_{tot}/f.u.) in terms of pressure, which determines the stable state under high pressure, the vdW results give the same conclusion as the PBE results. As shown in Supplementary Fig. 2 below, the vdW-calculated total enthalpy differences between the 3D-cubic and 1D, 3D-hex structures have the same trend as those in Fig. 2. The 3D-cubic structure becomes the most stable when the pressure is higher than 2.3 GPa. Both the calculations with and without vdW effect showed that the 3D-cubic is the most stable structure at high pressure.

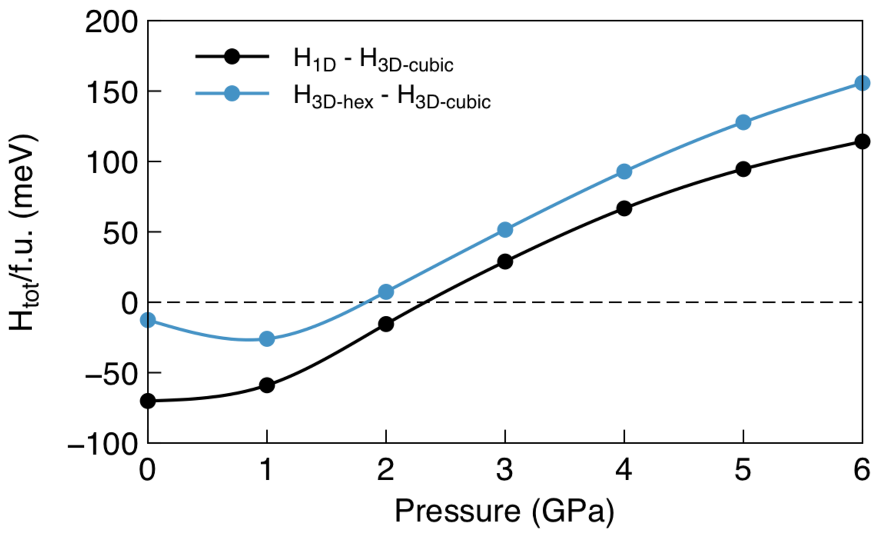

Supplementary Fig. 2 The H_{tot}/f.u. differences between the 3D-cubic and 1D, 3D-hex structures of $N(CH_3)_4SnI_3$ as functions of hydrostatic pressure. The vdW effect is included.

Supplementary Note 4. Energy cost of 3D-cubic $N(CH_3)_4SnI_3$ formation reaction

In order to confirm that the 3D-cubic $N(CH_3)_4SnI_3$ is stable with respect to the phase separation and is synthesizable under high pressure, we calculate the formation enthalpy (energy cost) of the formation reaction of 3D-cubic $N(CH_3)_4SnI_3$, $N(CH_3)_4I$ + SnI_2 → $N(CH_3)_4SnI_3$ [1], which was experimentally reported to occur at 120 °C [2]. The energy cost of the formation reaction E_f is defined as [3],

$$E_f = H_{\mathrm{N(CH_3)_4SnI_3}} - H_{\mathrm{N(CH_3)_4I}} - H_{\mathrm{SnI_2}}$$

where $H_{\mathrm{N(CH_3)_4SnI_3}}$, $H_{\mathrm{N(CH_3)_4I}}$ and $H_{\mathrm{SnI_2}}$ are the total enthalpies of 3D-cubic $N(CH_3)_4SnI_3$, $N(CH_3)_4I$ and SnI_2, respectively. The calculated energy costs under different pressures are shown in Supplementary Fig. 3.

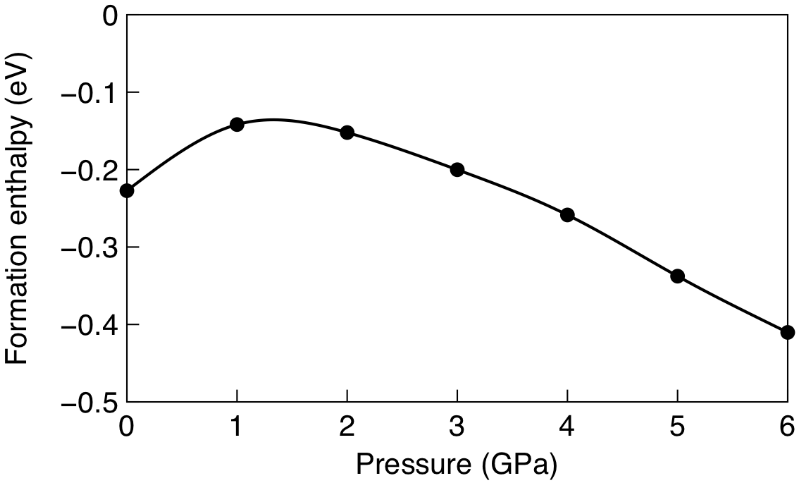

Supplementary Fig. 3 The energy cost of the formation reaction $N(CH_3)_4I$ + SnI_2 → $N(CH_3)_4SnI_3$ under different pressures.

Obviously, the energy cost E_f is always negative under the pressure 0-6 GPa, indicating that $N(CH_3)_4SnI_3$ is always stable with respect to phase separation into $N(CH_3)_4I$ + SnI_2. The energy cost becomes more negative as the pressure is higher than 1.5 GPa, indicating that the synthesis reaction is thermodynamically favored and the 3D-cubic $N(CH_3)_4SnI_3$ is synthesizable under high pressure.

Supplementary Note 5. Band structure of 1D $N(CH_3)_4SnI_3$

The band structure and density of states (DOS) of 1D $N(CH_3)_4SnI_3$ calculated using PEB+SOC are shown in Supplementary Fig. 4. The VBM is located at K (1/3, 1/3, 0) point and CBM is at Γ (0, 0, 0) point. The band gap E_g = 2.64 eV.

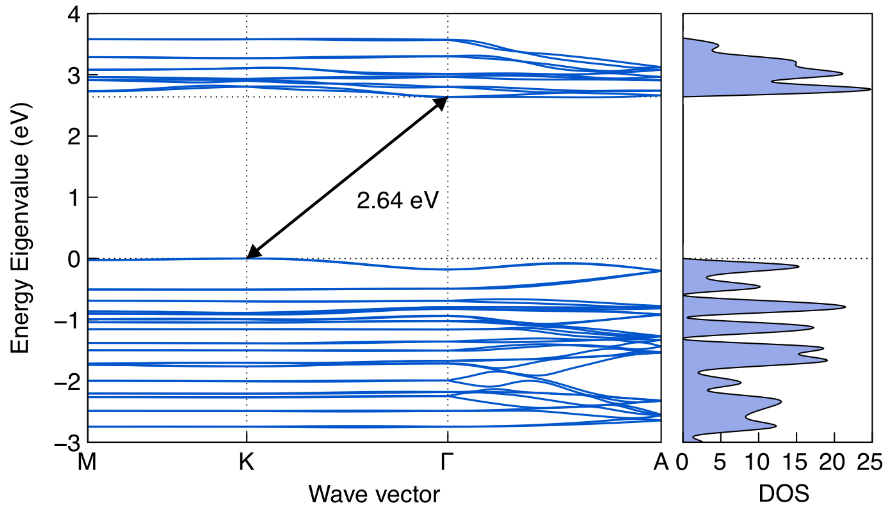

Supplementary Fig. 4 The band structure and DOS of 1D $N(CH_3)_4SnI_3$ calculated using PBE+SOC. The high-symmetry wave vectors are M (1/2, 0, 0), K (1/3, 1/3, 0), Γ (0, 0, 0) and A (0, 0, 1/2). The Fermi level is shifted to 0 eV.

Supplementary Note 6. Absorption coefficient of 3D-cubic $N(CH_3)_4SnI_3$

The light-absorber semiconductor in single-junction solar cells should have efficient absorption for the incident sunlight with energies ranging in 1.55–4.13 eV [4]. So we also calculate the absorption coefficient for 3D-cubic $N(CH_3)_4SnI_3$. Firstly, the imaginary part of the frequency-dependent dielectric function is calculated using the formalism given by Ref. 5 and the real part is derived using the Kramers-Kronig transformation. Then, the optical absorption coefficient can be calculated as following,

$$\alpha(\omega) = \frac{\sqrt{2}\omega}{c}\left[\sqrt{\varepsilon_{\mathrm{re}}(\omega)^2 + \varepsilon_{\mathrm{im}}(\omega)^2} - \varepsilon_{\mathrm{re}}(\omega)\right]^{1/2}$$

where $\alpha(\omega)$ is the absorption coefficient, ω is the angular frequency of the incident light, $\varepsilon_{\mathrm{re}}(\omega)$ and $\varepsilon_{\mathrm{im}}(\omega)$ are the real and imaginary part of dielectric function $\varepsilon(\omega)$, c is the light speed. The calculated $\alpha(\omega)$ is shown in Supplementary Fig. 5. The absorption starts from 2.1 eV which is the value of E_g and increases gradually as the incident energy increases up to 8 eV. Our calculated absorption spectrum satisfies the requirement of the light-absorber semiconductor in single-junction solar cells.

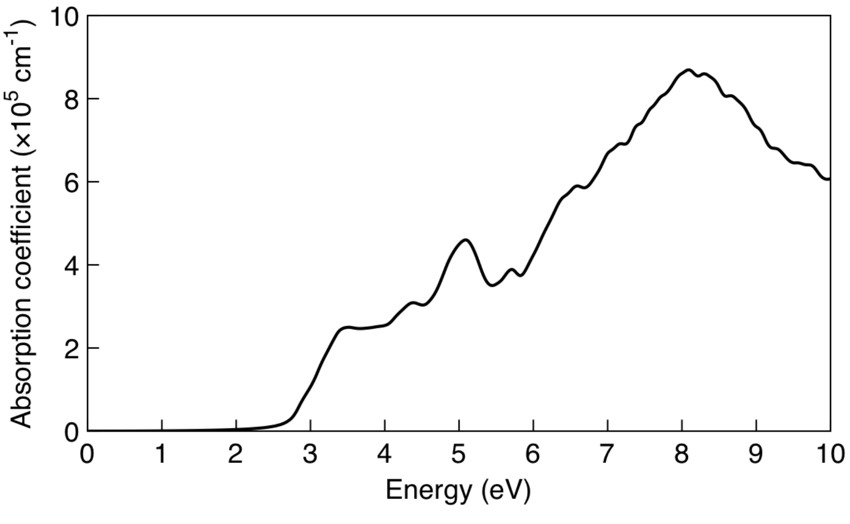

Supplementary Fig. 5 The optical absorption coefficient of 3D-cubic $N(CH_3)_4SnI_3$ at 0 GPa as a function of the incident sunlight energy.

Supplementary Note 7. Total energy of $CsSnI_3$ with different volume

The E_{tot}/f.u. of $CsSnI_3$ as a function of the supercell volume is shown in Supplementary Fig. 6. The equilibrium volumes/f. u. of $Pm\overline{3}m$ (Cubic), *Pnma*, *R3c* and *R3m* are almost the same, around 247 Å^3. The energy of *Pnma* is the lowest (see the inset in Supplementary Fig. 6), which means it is the most stable structure. As the volume increases, the total energies of all $Pm\overline{3}m$ (Cubic), *Pnma*, *R3c* and *R3m* structures increase. The increasing rate of *R3m* is the smallest. Therefore, the *R3m* structure becomes the most stable structure for the large volume.

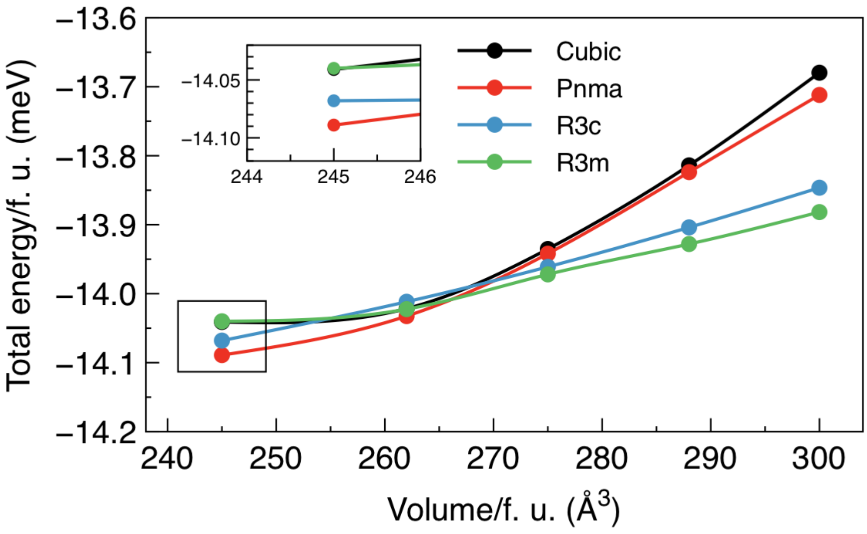

Supplementary Fig. 6 E_{tot}**/f.u. of** $CsSnI_3$ **as a function of the volume/f.u..**

References

[1] Soumyodeep Banerjee and R. N. Gayen, Tetramethylammonium based lead free perovskite active layer for solar cell application, *Ceramics International* **45**, 17438 (2019).

[2] Constantinos C. Stoumpos, Lingling Mao, Christos D. Malliakas and Mercouri G. Kanatzidis, Structure-Band Gap Relationships in Hexagonal Polytypes and Low-Dimensional Structures of Hybrid Tin Iodide Perovskites, *Inorg. Chem.* **56**, 56 (2017).

[3] Liyuan Wu, Pengfei Lu, Yuheng Li, Yan Sun, Joseph Wong and Kesong Yang, First-principles characterization of two-dimensional $(CH_3(CH_2)_3NH_3)_2(CH_3NH_3)_{n-1}Ge_nI_{3n+1}$ perovskite, *J. Mater. Chem. A* **6**, 24389 (2018).

[4] G. Tang and J. Hong, Direct tuning of the band gap via electronically-active organic cations and large piezoelectric response in one-dimensional hybrid halides from first-principles, *J. Mater. Chem. C* **6**, 7671 (2018).

[5] M. Gajdoš, K. Hummer, G. Kresse, J. Furthmüller, and F. Bechstedt, Linear optical properties in the projector-augmented wave methodology, *Phys. Rev. B* **73**, 045112 (2006).
